# Multi-Layer Blockchain-Based Security Architecture for Internet of Things

**DOI:** 10.3390/s21030772

**Published:** 2021-01-24

**Authors:** Houshyar Honar Pajooh, Mohammad Rashid, Fakhrul Alam, Serge Demidenko

**Affiliations:** 1 Department of Mechanical and Electrical Engineering, Massey University, Auckland 0632, New Zealand; M.A.Rashid@massey.ac.nz (M.R.); F.Alam@massey.ac.nz (F.A.); 2 School of Science and Technology, Sunway University, Selangor 47500, Malaysia; SDemidenko@Sunway.edu.my

**Keywords:** internet of things, blockchain, hyperledger fabric, evolutionary clustering, security, scalability, authorization

## Abstract

The proliferation of smart devices in the Internet of Things (IoT) networks creates significant security challenges for the communications between such devices. Blockchain is a decentralized and distributed technology that can potentially tackle the security problems within the 5G-enabled IoT networks. This paper proposes a Multi layer Blockchain Security model to protect IoT networks while simplifying the implementation. The concept of clustering is utilized in order to facilitate the multi-layer architecture. The K-unknown clusters are defined within the IoT network by applying techniques that utillize a hybrid Evolutionary Computation Algorithm while using Simulated Annealing and Genetic Algorithms. The chosen cluster heads are responsible for local authentication and authorization. Local private blockchain implementation facilitates communications between the cluster heads and relevant base stations. Such a blockchain enhances credibility assurance and security while also providing a network authentication mechanism. The open-source Hyperledger Fabric Blockchain platform is deployed for the proposed model development. Base stations adopt a global blockchain approach to communicate with each other securely. The simulation results demonstrate that the proposed clustering algorithm performs well when compared to the earlier reported approaches. The proposed lightweight blockchain model is also shown to be better suited to balance network latency and throughput as compared to a traditional global blockchain.

## 1. Introduction

Ubiquitous interconnected objects can be deployed through the Internet of Things (IoT) infrastructure using cloud platforms in a centralized network [1]. A wide variety of interconnected devices, including smart locks [2] and vehicles [3], can also implement decentralized solutions by employing the blockchain technology in a decentralized peer-to-peer manner [4]. Both of the models are capable of dealing with the challenges of providing privacy and security for networked devices in the IoT environment. Nevertheless, the constraints of limited resources, centralized control, scalability, overhead, latency, and throughput characterize the expected heterogeneity of IoT network devices [5].

In a centralized network structure, the server controls and enhances the performance of the devices [6]. However, centralized schemes have several drawbacks. The network with a large number of smart devices normally generates a tremendous volume of data. A cloud platform service-provider requires considerable network bandwidth as well as high-performance with regards to efficiency and storage [7,8]. Furthermore, there is always a risk of the centralized network key components failure leading to a serious (or complete) breakdown of the entire system [9,10]. The data that are collected by the central cloud storage often require further manipulation by a third-party. This potentially could lead to data leaks, thus compromising the end-user’s privacy [11]. The external computing resources coordination is another challenge for proving IoT security and performance in the centralized systems [9]. Therefore, most current centralized systems fail to provide entities with a guarantee of data reliability and privacy.

Most IoT devices are only able to communicate in short-range transmissions, due to their low-power wireless transmitters and receivers. IoT networks can benefit from utilizing the Multihop Cellular Network (MCN) concept [12] that facilitates significant shortening in signal coverage. The essence of MCN leveraged by distributed, decentralized blockchain technology can ensure the required high-security and credibility for the IoT network by addressing the drawbacks of the centralization servers [13]. Besides, it enhances the degree of trust between heterogeneous devices, and that can minimize the cost of conventional data-sharing platforms [14]. The formation of a large-scale network comprising of heterogeneous nodes is not as easy as traditional blockchain implementation needs high-performance nodes. A self-protection mechanism is also required, due to the distributed structure of IoT networks with a multitude of objects and devices [15].

The increased number of connected devices (in the order of million devices per sq km), heterogeneity of devices and vendors, interoperability, a vast amount of collected data and network traffic, requirements of large bandwidth capacity, communication latency, and trust are the major challenges within the new era of the 5G-enabled IoT. [16]. The new model of security should address the unique requirements of the 5G-enabled IoT and D2D (Device-to-Device) communication devices such as scalability, low latency, energy concerns, secure communication, and reliability. Blockchain technology, including bitcoin [17,18], have been implemented for security enhancement for a long time. Their technical value has been generally recognized. At the same time, their functionality support is still limited to simple transactional data storage. Furthermore, the blockchain is a viable option for supporting ultra-reliable low latency massive Machine Type Communication (mMTC) of resource-constrained IoT devices under the 5G networks for improved security and privacy.

This paper discusses a multilayer architecture that is based on a new clustering model suited for blockchain implementation to tackle the issues associated with implementation complexity and elaborate on the mechanism for securing IoT communication. The new network model that is based on multi-layer distributed blockchain can be regarded as an organic combination of the blockchain technology and clustering techniques that effectively utilize network clustering performance and capabilities, and significantly improve the overall security and reliability of the IoT. An adapted clustering algorithm has been developed to suit the IoT systems’ blockchain implementation by considering the network performance metrics in defined cost functions. The IoT network clustering aims to reduce the network load, enhance coverage, and minimize the energy (as reflected in the distance) while leveraging the essence of MCNs. The multi-layer structure facilitates the detection of compromised entities within the entire network in each layer. Each transaction in the system needs to be verified by other participants by implementing a consensus algorithm. Blockchain is continuously monitored by the entire network participants, maintaining a copy of the blockchain ledger. Therefore, compromised nodes have no means of inserting fraudulent blocks into the public ledger without immediately being noticed by others. Thus, the multi-layer blockchain removes compromised entities from being a part of the system. This makes it impossible to compromise the integrity of records in the blockchain. Another crucial point is that the new multi-layer architecture allows for upgrading for the existing central cloud server. This makes large-scale deployments possible. Besides, a lightweight authorization and authentication process running in each cluster guarantees secure access to the network resources through implementing smart contracts.

The rest of the paper is organized, as follows. In Section 2, a literature review on the blockchain implementation in the IoT environment is introduced along with essential information on the blockchain and IoT security. Section 3 details the framework architecture and multi-layer system. Section 4 provides the proposed IoT blockchain framework implementation and associated results. The proposed clustering algorithm is based on the Genetic Algorithm (GA) and Simulated Annealing (SA) [19]. Section 5 illustrates the challenges addressed by implementing the proposed system model. Finally, Section 6 draws a conclusion and presents future research directions.

## 2. Related Works

Fast-growing numbers of networked devices characterize modern IoT systems. Consequently, the amount of generated data by the connected devices is also escalating. This inevitably leads to security and privacy concerns. Security (along with computing and communication issues associated with IoT devices) is mainly due to the limited memory capacity and processing power of the devices [10].

### 2.1. Authentication and Authorization in IoT

Devices require authentication and authorization to enter the IoT system. These measures are considered as a critical juncture of network security [20]. Interconnected devices within the IoT environment are required for establishing secure communication with the aid of relevant authentication procedures. The authentication and authorization processes of the interconnected nodes and devices are traditionally maintained by a central authority in the IoT network based on the Public Key Infrastructure (PKI) [21]. Therefore, the process increases the authority center’s workload significantly and it causes considerable delay due to a large number of requests [22]. To this end, several new authentication models have been proposed. The method that was proposed in [23] for the authentication and privacy is built up upon IP-Sec and Transport Layer Security (TLS). However, such a mechanism is not suitable for resource-constrained interconnected IoT devices due to the high demand for computational resources.

Research [24] develops an access management mechanism that is based on blockchain decentralized architecture in the IoT system. The proposed approach eliminates the centralized control server and implements the Proof of Concept (PoC) as a consensus algorithm. The development of a secure access control mechanism for IoT is presented in [25] in order to address the issues related to the distribution of access rights delegation. This approach uses the blockchain Ethereum technology to validate the identity of the entity. Research [26] proposes a framework with layers, intersect, and self-organization Blockchain Structures (BCS) to verify IoT entities. Model efficiency and security performance are analyzed in terms of storage efficiency, response time, and verification. Paper [27] highlights the concerns that are related to privacy and security of data authentication in IoT. The blockchain technology has been seen as a potential fabric for eliminating the central server concept, and distributed futures helps to address IoT challenges, such as device spoofing, false authentication, and lower reliability in data sharing. The authors in [28] propose a structure for security and authentication in IoT that is based on the blockchain. This proposal addresses the single-point-failure issue.

### 2.2. Blockchain-Based Frameworks for IoT Security and Privacy

Researchers have been developing blockchain technology to address the privacy and security challenges in the IoT as an alternative solution. The implementation of several privacy preservation strategies in blockchain-based IoT systems is discussed in [29]. These strategies include encryption, anonymization, private contract, mixing, and differential privacy. The authors of the research [30] review the blockchain technology and applications for IoT systems as well as a way the blockchain techniques can address the security challenges within the IoT systems. The lack of a comprehensive standard architecture, cloud server availability, capacity, susceptibility to manipulation, and cost limitations are highlighted as the critical challenges with the blockchain technology implementation in IoT [7,8].

Lightweight Scalable Blockchain (LSB) is presented in [31] in order to facilitate the privacy and security of the IoT devices. An overlay network is proposed to achieve decentralization and maintain end-to-end security and privacy with the blockchain-based framework implementation run by devices with robust computation capabilities. A new Proof of Block and Trade (PoBT) consensus algorithm is proposed in [32] in order to address the challenges associated with integrating salable IoT networks and blockchain technology. The research aim is to reduce the computation time for the validation of trades and blocks. The work is also considered a ledger distribution mechanism to reduce the memory requirements of IoT devices. The study that is presented in [5] suggests using LSB to build the blockchain-based model on the modified consensus algorithm to minimize the Proof of Work (PoW) deployment complexity. Hence, the author replaced the PoW with a distributed trusted consensus algorithm. The proposal enhances the privacy and security of IoT networks in a decentralized manner. The research in [33] proposes a blockchain-based framework to address privacy, security, fault-tolerance, and autonomous behavior issues. The framework helps to assess the possible blockchain implementation through a decision structure for IoT and edge computing.

Data operations are performed in the blockchain system through smart contract implementation, including data gathering, invoking, transfer, storage. A new context-aware mechanism is proposed in [34] for blockchain-enabled IoT systems to facilitate the on-chain data allocation. The authors define a fuzzy logic mechanism to control the data and calculate the Rating of Allocation (RoA) value that is associated with each data request. The efficiency of the proposed mechanism is investigated in the blockchain-based cloud and fog architectures implementations.

### 2.3. Permissioned Blockchain in IoT

Hyperledger Fabric (HLF) [35], which is a distributed ledger technology, paves the way to leverage a trustful environment without central authority dependency while delivering a high degree of flexibility, scalability, and confidentiality. The consensus algorithm is an open architecture in HLF. It provides a flexible environment for modifying the configuration and increase the performance. A new authorization framework for an IoT network is proposed in [36] based on the HLF framework. The work focuses on enhancing the consensus algorithm by implementing the GA optimization. The aim is to attain the best configuration with input transactions and success rates as input parameters to the GA algorithm. The IoT data management and its traditional characteristics have been considered in [37]. The research proposes a permissioned blockchain-based decentralized trust management (BlockBDM) in order to address the security and trust problems of IoT big data management.

### 2.4. Layer-Based IoT Blockchain

A platform for facilitating secure communications for smart cities is proposed in [38]. The presented solution deploys a layer-wise security structure by integrating smart devices and blockchain technology. Paper [39] has proposed a multi-layer IoT blockchain-based solution that is specifically modelled for use in the medical field. The solution addresses computation and complexity issues of the blockchain implementation by converting IoT networks into decentralized multi-layer structures. The research presented in [40] proposes a hybrid network architecture for the smart city by leveraging the strength of emerging Software Defined Networking (SDN) and blockchain technologies. In order to achieve higher efficiency, the proposed architecture is divided into two parts: the core network and edge network. This model inherits the strength of both the centralized and distributed network architectures. In [41], the authors proposed a multi-level blockchain framework to enhance privacy and data security in IoT applications. The multi-level model focuses on improving the response time and resource utilization. The authors define mobile agents to perform the hash function, implement encryption, deploy aggregation, and decryption. The mobile agents are transferred between blockchain and IoT in order to accomplish the required tasks. A two-tier hierarchical blockchain framework for IoT is proposed in [42] for enhancing and measuring the scalability of a blockchain application in a IoT car rental system.

Some of the previous works discuss the multi-layer based blockchain approach for the integration of IoT and blockchain technology. The multi-layer based blockchain network model is introduced in [43] in order to overcome the challenges of conventional centralized network architecture. The proposed model reduces the difficulty of the blockchain deployment in IoT systems by dividing the network into a multi-level decentralized network. Hybrid IoT [44] is a new hybrid blockchain platform for IoT. It is based on the implementation of PoW and Byzantine Fault Tolerance (BFT) consensus algorithms. The proposed structure includes sub-blockchains and inter-blockchains. The BFT inter-connector platform connects two PoW sub-blockchains. An integrated blockchain-IoT is proposed in [45] in order to secure the digital system for healthcare. The work addresses the scalability challenges in the IoT system.

In [46], the authors proposes a double-chain (alliance and private chain) model that considers the IoT environment for the data-sharing-transaction application. In the multi-layer model, the alliance chain processes the transactions. The transaction data record in the blockchain ledger is performed by the private chain that is deployed within each organization. The real blockchain system data is stored in an IPFS cluster server built by the alliance stores. Paper [47] proposes a hierarchical resource allocation framework based on the blockchain for edge computing. The presented model implements a smart contract-based hierarchical auction mechanism for solving resource allocation challenges for the IoT devices that are located beyond the coverage of Access Points. A blockchain-based multi-layer hierarchical architecture proposed in [48] facilitates the monitoring and managing of the Internet of Underwater Things (IoUT) on cloud data. Sensor nodes are clustered and organized based on selected residual energy cluster heads. The cluster head and node tracking are performed by using the Bloom filter. The gateways communicate by deploying a standard secret key, separated from another secret key that is used by the cluster head. Subsequently, the blockchain ledger stores the routed data. A fog layer smart gateway merged into the IoUT blockchain addresses the transaction preparation challenges, data routing to miners problems, and scalability issues [49]. The proposed model deploys a lightweight consensus mechanism to add blocks in the blockchain where the IoUT data are stored.

Unfortunately, the majority of the solutions proposed in the literature do not address the problems that are associated with the implementation of the blockchain technology in IoT systems, such as device authentication, low scalability, transaction delays, high computational resources for mining, and device heterogeneity. In our previous work [50], some of the challenges that are mentioned above are highlighted along with the discussion on the adoption of the blockchain technology in the IoT context. This article expands the implementation of the Lightweight Hyperledger Blockchain (LHB) technology and smart contracts to enhance the performance of the blockchain-IoT combination.

The heterogeneous IoT network lifetime improvement is achievable by implementing a clustering model along with a multi-layer structure. The clustering concept is the key to achieving the multi-layer architecture, where the cluster heads form the multi-layer structure. Clustering techniques for wireless networks and device-to-device (D2D) communications systems have been widely reported in the literature. They offer reduced energy consumption and higher throughput [51]. A self-clustering method is proposed in this work in order to identify Cluster Head (CH) nodes. Genetic algorithms considering various clustering factors, including geospatial ones (e.g., the distance between nodes, the base-station distance to nodes) and total network energy, are proposed. A fitness function simulating network changes and node movements within the network is optimized by deploying the SA methodology.

In the multi-layer architecture, devices in each layer have different computational capabilities and energy storage capacity. Consequently, different security strategies are proposed for individual layers. Each design is based on the blockchain. Even so, the blockchain implementation is modified to suit the devices of each particular layer. The key contribution of this research is three-fold:

1. A novel, lightweight, private multi-layer model is proposed for reducing the complexity of blockchain technology implementation while improving the network scalability. The proposed model is tailored to meet the requirements of IoT devices by adopting the blockchain technology to suit different layers of the IoT system. The simulation study shows that the proposed Hyperledger Fabric-based method outperforms a traditional blockchain solution, like the Ethereum, in terms of latency and throughput.
2. Clustering is one of the key steps of implementing the multi-layer architecture. Therefore, a new network clustering method is presented. It is based on the evolutionary computation that deploys multi-objective fitness functions that are relevant to heterogeneous IoT networks. The decentralized, fast, and self-clustering method divides the IoT network into clusters while considering the node mobility. The simulation results show that the proposed clustering algorithm outperforms existing solutions.
3. A novel method of authentication and authorization of IoT nodes is implemented in order to provide security for IoT devices and protect device communications through a multi-layer structure.

## 3. Multi-Layer Security Framework

The aim of the proposed network model is to provide a reliable trustful security mechanism for IoT networks while using the performance and capabilities of the cellular system. The intelligent clustering and machine learning approach based on Swarm Intelligence (SI) and Evolutionary Computation (EC) algorithms [19] is deployed in order to encode the multi-layer structure. This proposal provides a framework to facilitate the lightweight authentication and authorization of IoT networked devices (objects and nodes) based on the blockchain technology.

**Figure 1:**
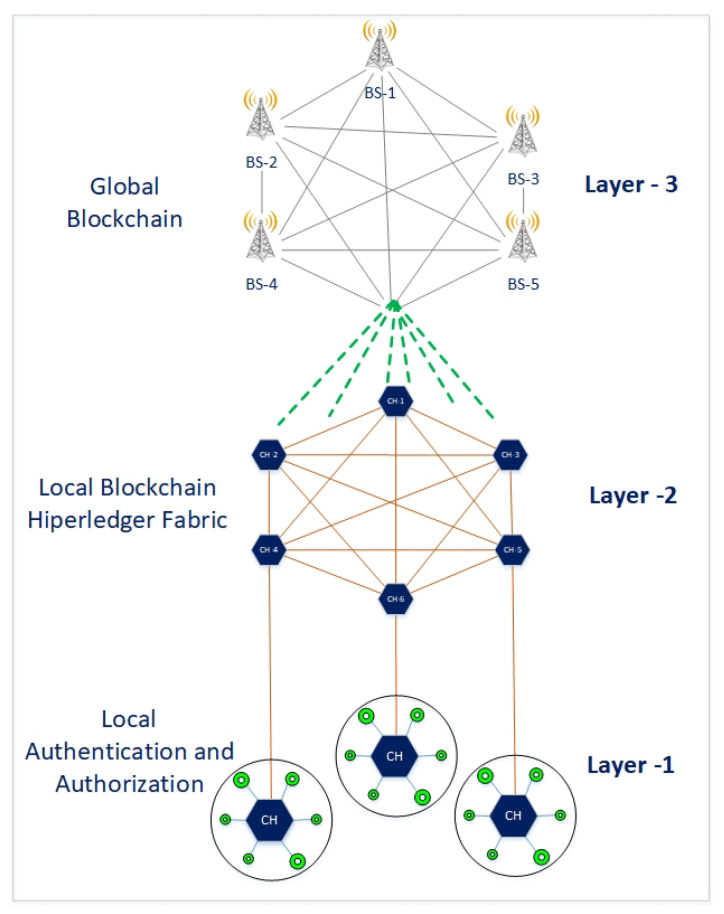
Multi-Layer model for Internet of Things (IoT) network.

The proposed multi-layer network model divides the entire cellular-enabled IoT network into multiple tiers. Layer-1 consists of various clusters and IoT nodes. Layer-2 includes sink nodes and controlling devices, such as cluster heads. Layer-3 contains the base stations of a cellular network. All of the CHs, as cellular devices, have cellular connectivity with the 5G BSs, and, thus, via the BSs/D2D capability, also with other CHs. The BSs have the processing power (with appropriate servers and CPUs) to implement the decentralized blockchain mechanism at Layer-3. Figure 1 shows the overall system model.

**Figure 2:**
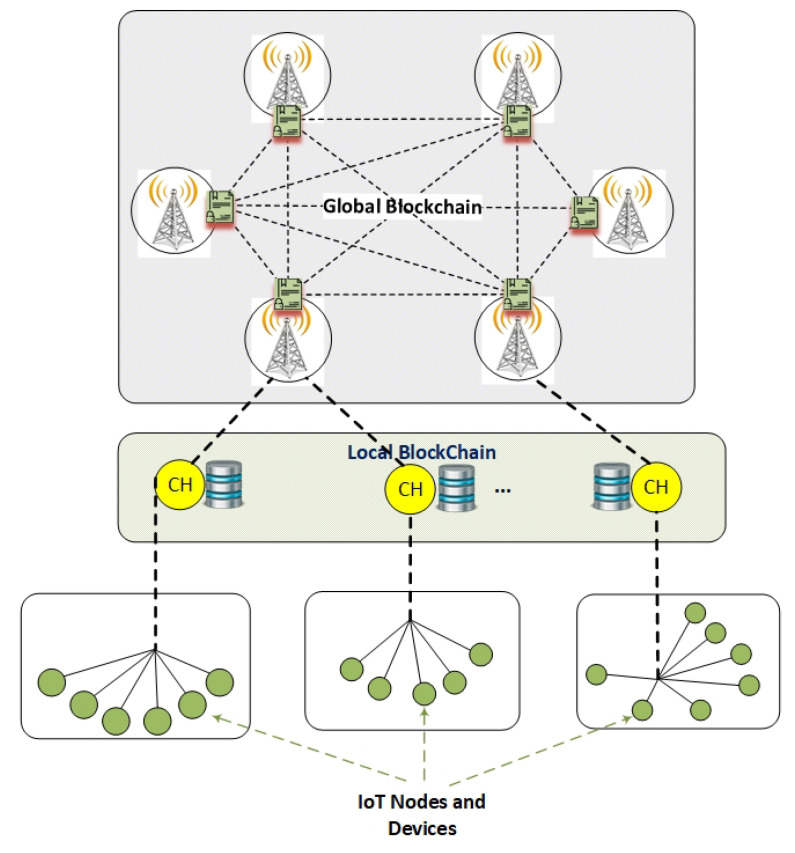
The network model based on a multi-layer structure that implements a local authorization service in the infrastructure level, a local blockchain, and public chain in the remaining two layers.

Blockchain implementation can potentially lead to additional overhead and scalability issues [5]. The multi-layer network model, as shown in Figure 2, is proposed for minimizing the overhead, reduce delays, and response time, create associated channels to collect specific data, secure communication, and address the need for the network scalability. The first layer contains devices and nodes with a diverse range of computational capabilities and power resources. Locally registered devices use authentication and authorization services through a local authorization program that is run by the cluster heads in the IoT network. The second layer includes CH nodes, authority nodes, edge-computing nodes, and gateways. CH nodes can securely communicate in the blockchain environment that deploys a lightweight consensus. The local permissioned HLF blockchain is implemented in this layer. The last layer consists of BSs in cellular networks. This higher layer, which consists of resources with high computational power, can be arranged as a set of separate structures under the HLF blockchain [52]. Robust asymmetric cryptography mechanism deployment can be achieved in this layer. The security and privacy are guaranteed with the implementation of the Global Blockchain and sophisticated security approaches to the high-level layer (Layer 3).

### 3.1. LAYER-1

This level includes IoT objects, nodes, and devices, as well as network elements for communications, network procedures, and protocols. Unsupervised hybrid clustering algorithms, as described in Section 4, convert the entire IoT network into multiple clusters and to form the layers. Each cluster is associated with a powerful device chosen as CH. IoT devices and nodes are geographically distributed non-uniformly. The devices are authenticated and authorized to the network through a local authorization and authentication services to guarantee the privacy and security inside each cluster.

The intra-cluster security and privacy are facilitated by local CH nodes acting as edge processing nodes, as shown in Figure 2. Such an approach can enhance the implementation efficiency while also addressing the issues that are associated with globally centralized cloud computing.

A lightweight session key is assigned to devices when they authenticate to associated CH nodes and establish communications. The session key period validation is carried out by the cluster heads in order to perform the authorization and authentication. The registration services and authentication management, as well as authorization, are also locally performed by the CH nodes to improve scalability and address device heterogeneity. CH nodes maintain the addition of new devices to the network through a local registration process. The cryptographic key distribution or session keys allow the node authentications. Less power-hungry cryptography is provided by edge computing. Alternatively, CH nodes for IoT devices with limited resources could provide long-term cached session keys (cryptographic keys).

Lightweight session keys are assigned by CH nodes in order to maintain the authorization of registered nodes as an authorization entity and authenticate them to the network. Symmetric keys and lightweight cryptography are proposed to tackle the scalability challenges and the limitation of IoT devices with constrained resources. CH nodes perform the following four tasks:

- a new node registration to the network as a new entity;
- session key (cryptographic key) distribution and assignment;
- communications management and initiation; and,
- secure communications management and establishment.

**Figure 3:**
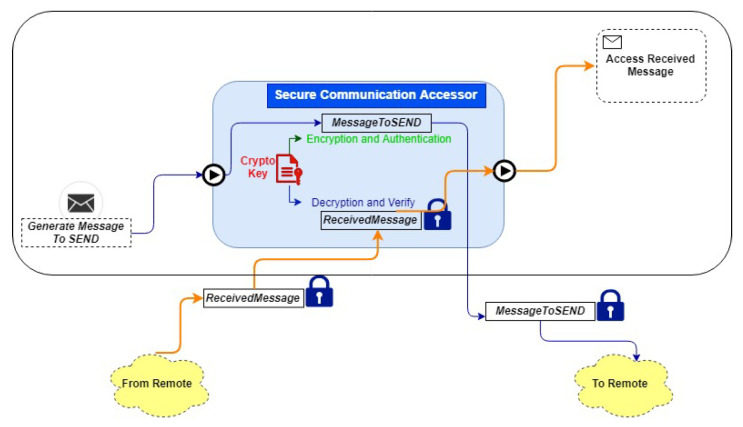
Local authorization service among IoT entity and Cluster Head (CH) nodes for secure communication.

Symmetric key-wrapping encrypts the lightweight session keys, called the distribution keys. Every single communication is protected with a session key. The session key is a symmetric key that has a unique ID and a period of validity. The use of cryptographic keys (credential management) for encryption, message authentication, and decryption is managed by secure communication. Consequently, selected CH nodes are responsible for managing cryptographic keys. Figure 3 illustrates the overview of the authorization procedure.

### 3.2. LAYER-2

The second layer connects all of the selected CH nodes under the serving Base Station (BS) units. Cluster heads collect and forward data to the higher layer. All of the nodes in the second layer run a private LBC in a distributed manner to achieve a consensus that is based on the defined consensus algorithm [5]. BS and CH units generate and verify blocks, handle communications with non-consensus devices and nodes within the same layer, and broadcast blocks to each cluster. Trusted nodes provide interfaces to subordinate and superior layers employing the blockchain protocol. The HLF blockchain platform is proposed for this layer.

The proposed network model needs to consider secured CH communications while addressing the resource-constrained and decentralized node distribution of the IoT network. The blockchain mining process is computationally intensive and time-consuming. Hence, it is not ideally suited for implementation in an IoT system. Therefore, the proposal suggests a lightweight, private, decentralized blockchain-based method for data communications built upon distributed consensus. It is important to consider the limitation (resource-constraint) of devices in the IoT system. Thus, a lightweight cryptographic mechanism is to be implemented.

In the proposed model, the interaction between CH nodes and other networked elements are based on local permissioned HLF blockchain [53] platform. HLF is a private permissioned blockchain and it is based on an execute-order-validate architecture. In this architecture, the transaction execution (via smart contract) is separated from transaction ordering in order to achieve better scalability and modular consensus implementations.

**Figure 4:**
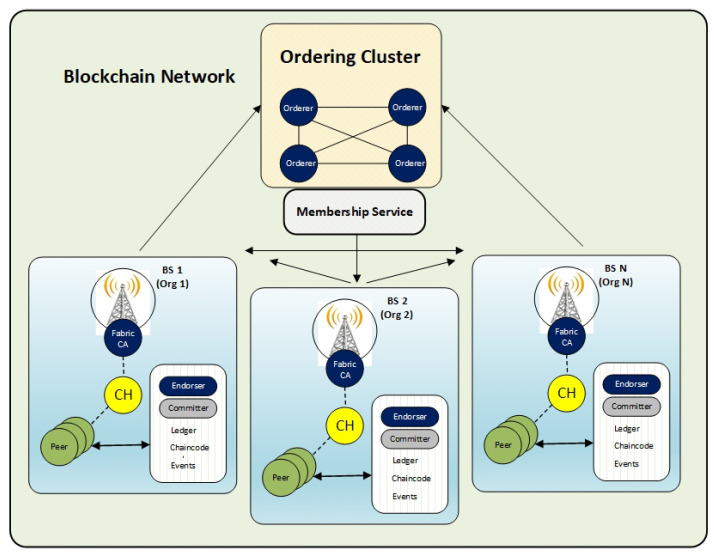
The network architecture of the IoT-enabled cellular system combined with the blockchain technology and smart contracts.

The main elements of the proposed model are organizations (base stations), IoT nodes and objects, ordering clusters, and peers (including endorser and committer, membership service providers (MSPs), and channels [54]), as shown in Figure 4.

The peers maintain distributed ledger and execute transactions. A peer node can be an endorser or committer or both. The orderers are responsible for ordering all of the transactions in the network. In addition, orderers propose new blocks and seek consensus. Ordering service is a collection of orderers. All peers are committers by default. Ordering service sends the ordered state updates in the form of a block of transactions, and committers maintaining the ledger. The peer validates transactions of a new block, commits the changes locally as a copy of ledger, and updates the blockchain by appending it on the block. Peers also can be an endorser for endorsing transactions. An endorser executes the smart contract (ChainCode in HLF) and appends the results with its cryptographic signature (called endorsement) before sending it back to the client. In the proposed model, CH nodes can take the endorsers or committer roles, based on predefined roles.

MSP carries out the authentication services in the Hyperledger network. MSP has to verify the network nodes identity. The organizations are the logical representation of the Hyperledger framework. They are responsible for the management of the network members with the help of MSP. Channels facilitate the various connections within the network between its different elements while using private or dedicated channels. The committers perform the validation and update of the shared ledger.

The Hyperledger Blockchain is implemented through various transactions for data collection and data transmission. Transactions are defined using smart contracts [54]. Base stations in the high-level layers provide an organization for the blockchain implementation. They are connected to CH and different nodes in the IoT system. The ordering cluster handles the transactions and queue orders while providing a shared channel for different peer-to-peer communications. Additionally, the ordering service performs messages broadcasting, including transactions, and creating transaction blocks. IoT devices send transactions to ordering clusters while using Ordering Service (OS) to make a block of transactions. Defining the IoT nodes in the blockchain network to have an endorser or committer role depends on various metrics, e.g., the network configuration. Aside from validation tasks and updating the blockchain state, the committer node is responsible for block addition to the blockchain ledger.

An IoT node becomes an endorser through submitting an endorsement request. This request is sent to the endorser node for approval and consistency monitoring. The process of consistency check proceeds with the smart contract execution. The endorser sends back the response to the associated IoT node requests and grants a specific read and writes access.

The transaction block creation is performed by ordering clusters through the OS. The transaction blocks are distributed to all CH nodes. The blockchain system in this level updates the ledger, and transactions are added to the ledger along with IoT node specifications. A copy of the Blockchain ledger is shared with all of the peers in the network after validation.

### 3.3. LAYER-3

This layer consists of a distributed networked collection of BS nodes acting as an organization owner. Base stations manage devices, generate data, and process requests in a cloud server manner. The trusted nodes in this layer have powerful computing resources with less power and processor limitations. Consequently, more robust asymmetric cryptography mechanisms are proposed for this level with the aid of the global blockchain.

The high-level layer consists of BS nodes that can perform independent mining tasks without reliance on the central authentication servers. The nodes in this layer are computationally powerful while forming a distributed network topology. Therefore, deploying a suitable global chain, such as the global Ethereum blockchain framework, along with more sophisticated security techniques is feasible. The deployment of asymmetric cryptography, such as Elliptic Curve Cryptography (ECC) [55], is an appropriate solution for this layer. The blockchain-based system implementation enhances the level of privacy and security while guaranteeing data integrity. The higher layers do not include any central node, while the devices are data independent. The blockchain network records the transaction exchange between the nodes of this layer. The cluster heads, base stations, and computing edge nodes initiate the globally distributed trust relationship service mechanism among other network members.

The peer-to-peer nature of the blockchain provides a suitable solution for a globally distributed security framework between different network entities, such as BS nodes. The communication among CH nodes and computing edge nodes is done through implementing the blockchain-based communication with the use of certificates. Smart contracts maintain the distribution of the certificates to perform a trustful communication within the blockchain system among different nodes in this layer. CH nodes are required to sign the certificates. The proposed blockchain-based model is enhancing the distributed trust between two CH nodes and related BS nodes when they collaborate for authorization of their entities. It also enhances the trust, while an entity or IoT node establishes communication with other nodes in separate clusters under each BS.

The blockchain-based system maintains smart contract execution in order to avoid the requirement of using domain names and fixed addresses while the nodes establish the communications. The fixed addresses and domain names are not needed for the cluster heads in the proposed model for their communications with the edge devices as well as to execute smart contracts.

## 4. Framework Implementation

### 4.1. Network Self-Clustering

**Figure 5:**
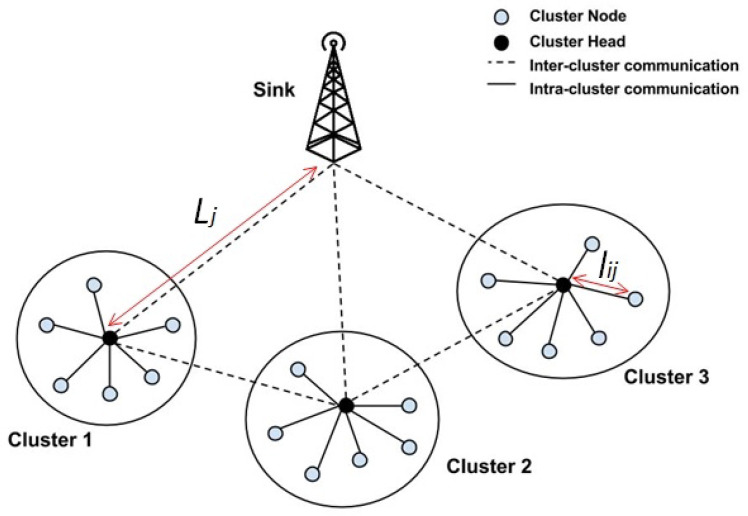
Network clustering scheme for cellular IoT network.

**Figure 6:**
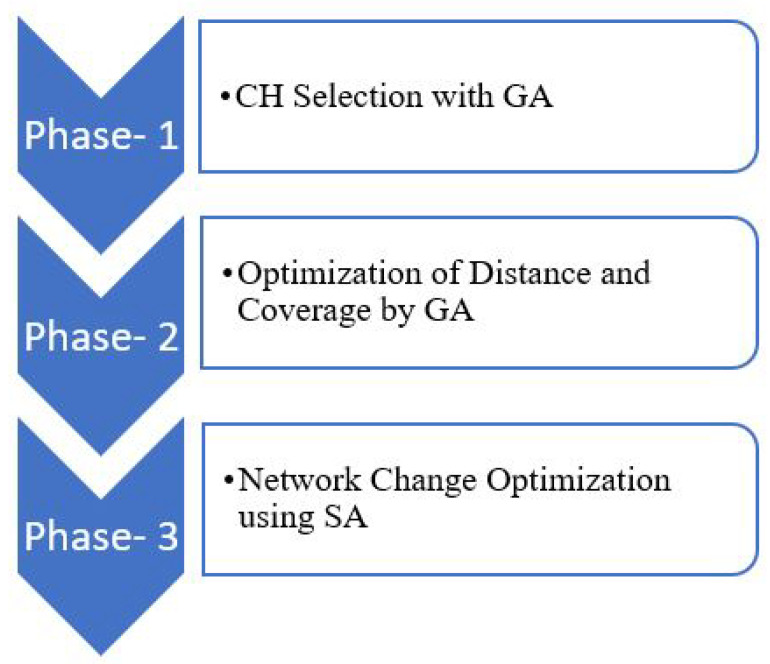
The flow of the Genetic Algorithm-GA-Simulated Annealing (GA-GA-SA) clustering algorithm.

Figure 5 shows the clustering approach for the IoT network. Figure 6 shows the flow of the proposed network clustering algorithm. It can be seen that the clustering is done with the utilization of metaheuristic algorithms [56]. Metaheuristic algorithms are based on a close interaction between computational practices and optimization. The main advantage of these methods is that they are unentrapped in local optimal points [57]. Therefore, these approaches seek all over the entire search space. Furthermore, the control within the metaheuristic algorithms is fully distributed among individuals (network nodes and participants). These individuals communicate with each other in a localized manner. The system response is robust, and the application for environment changes is fast [56,57,58].

GA is a population-based algorithm that is a subset of metaheuristic methods. It shows a good global-based exploration performance for the search of a problem space [59]. Therefore, GA is proposed in this research for the heterogeneous IoT network clustering. Furthermore, a good local-based exploration mechanism within the search space is required in order to evaluate a single solution. While SA indicates very good performance in this manner, a hybrid mechanism (built upon GA and SA) is chosen in this paper to optimize the proposed IoT network clustering [60].

The clustering approach also reduces the latency and overhead in the IoT systems via the overall minimization of communication distances among IoT objects and selected cluster heads. With clustering, a lower number of nodes require long-distance transmissions to BS nodes. Therefore, the total energy consumption for the entire system is reduced, while the network coverage is enhanced [59,60,61]. The clustering-based approach helps to leverage the blockchain technology application efficiency by reducing the deployment complexity. The entire network is divided into non-overlapping clusters that are managed by the CH nodes. Other cluster members communicate with the CH nodes for data transmission.

The idea is to achieve the clustering through the deployment of the evolutionary computation algorithms within the network. In the proposed model, critical network attributes, such as the distance, network coverage, energy, and load, are the parameters considered for the clustering of the nodes.

A hybrid algorithm (consisting of a Genetic Algorithm and Simulated Annealing) is employed for the selection of a cluster head as well as for cluster optimization, as shown in Figure 6. The proposed self-clustering approach allows for avoiding a uniform distribution of nodes and clusters. This is done to model the heterogeneous nature of the IoT network. The total number of clusters as well as the number of nodes that belong to each cluster are not predefined. Besides, the proposed clustering enhances the flexibility of nodes deployment in the IoT network. The nonuniform distribution of nodes in each cluster is considered. Consequently, the lifetime of the entire network increases, while the energy dissipation among the CH nodes is more uniform.

#### 4.1.1. **GA Phase:** CH Selection with Genetic Algorithm

The most critical factor in the IoT network design is satisfying the energy constraint. Longer network operation can be achieved through the shortening of communications and transmission links as well as by reducing the power consumption. Shorter communication links are achievable by grouping nodes into independent clusters. Such an approach facilitates the aggregation and forwarding of data, because each cluster member needs to exchange its information with the associated CH. The calculation of energy consumption uses the first order radio communication model [62]. The radio energy dissipation for transmitting or receiving a bit of data is equal to $E_{elec}$. Energy dissipation for transmitting *n* bits of data from the transmitter to the receiver node at the distance *l* can be calculated, as follows [63]:

(1) $$E_{tx}\left(n,l\right)=E_{elec}\times n+E_{amp}\times n\times l^{2},$$

while the energy dissipation volume in a node to receive *n* bits of data is formulated as:

(2) $$E_{rx}\left(n\right)=E_{elec}\times n,$$

where, $E_{elec}$ is the dissipation of radio energy and $E_{amp}$ is transmission amplifier energy dissipation.

Consequently, the total energy requirements to send and receive *n* data bits between two nodes located at the distance *l* comprise two main elements. The first component is the energy for amplifying data, transmission, and receiving. The second component includes the energy for the data processing by the node. The current leakage is considered to be negligible in low voltage and high-frequency systems. Equation (3) denotes $E_{ll}$ the total energy loss for the distances that are shown in Figure 5.

(3) $$E_{ll}=\sum_{j=1}^{k}\sum_{i=1}^{m_{j}}\left[l_{ij}^{2}+\frac{L_{j}^{2}}{m_{j}}\right],$$

where, $L_{j}$ represents the distance between the cluster-head and computing edge node; the $l_{ij}$ represents the distance between a node and its related cluster head (Figure 5); *k* represents a number of cluster heads; and, *m* represents the total number of nodes on the network.

Clustering is performed by considering the node residual energy, node distance from the BS, number of CH nodes, and CH distance from the other cluster heads (intra- and inter-cluster distances).

The cost function for the optimization problem considers the total transmission distance as a key metric that is to be minimized. Furthermore, the fitness function also takes the number of cluster heads into account while optimizing the network load. The following multi-objective cost function evaluates each individual node in the GAs algorithm: 

*Minimize:*

(4) $$cost\left(f_{1}\right)=\omega_{1}\left(\frac{E_{dd}}{D}\right)+\omega_{2}\left(\frac{CH_{i}}{m}\right)+\omega_{3}\left(\frac{Load}{m}\right),$$

where, $E_{dd}$ is the sum of CH distances to all individual nodes and the sum of the computing edge node distances to all CH nodes, $CH_{i}$ indicates the number of cluster heads, *D* is the network scaling dimension, *m* is the total number of nodes, *Load* is the max network load, and $\omega_{1}$, $\omega_{2}$, and $\omega_{3}$ are predefined constant weights.

**Figure 7:**
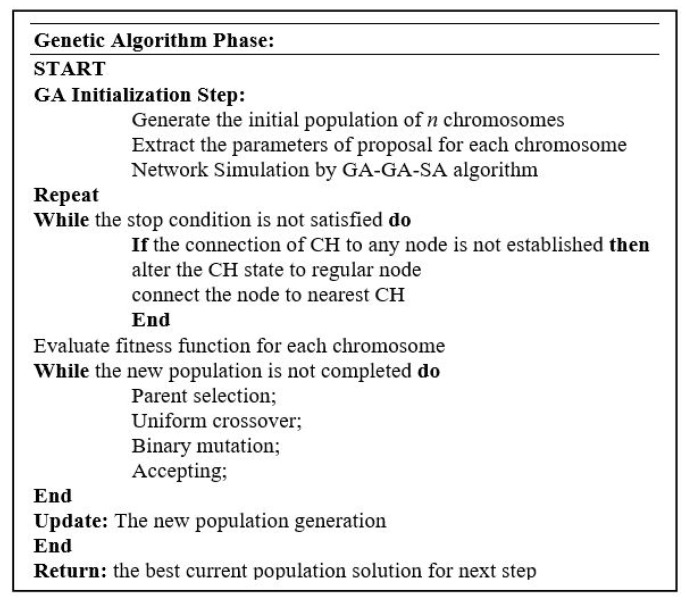
GA algorithm pseudocode.

The goal is to attain a fewer number of CH nodes and enhance the energy. The weights $\omega_{1}$ to $\omega_{3}$, with values between 0 and 1, represent the importance of the key metrics during the optimization procedure. Their values are chosen according to the importance of cost function factors [60]. GA minimizes the cost function at this stage. Figure 7 shows the initial phase of the clustering algorithm.

#### 4.1.2. Optimization of Distance and Coverage by GA

The GA approach helps to achieve distance optimization through its self-organized feature. The next GA starts with the final global solution of the first step GA as the initial solution, as outlined in Figure 6. This step can effectively formulate the mobility of different nodes. The proposed GA method also takes network coverage optimization into account.

The second GA phase enhances the GA solution of the first phase by a local search strategy. Optimizing the distance between CH to a node, and the CH to the sink or edge computing node results in the minimization of the total network energy dissipation. The distance-based equation is deployed in order to cluster the nodes in multi groups in the previous step and define the number of clusters through implementing the GA algorithm. The initial population for the current stage is generated from the best solution of the last phase. A multi-objective cost function is used in the GA optimization step. The distance is optimized while maximizing the coverage: 

*Minimize:*

(5) $$cost\left(f_{2}\right)=\omega_{4}\left(E_{ll}\right)+\omega_{5}\left(1-Coverage\right),$$

where, $E_{ll}$ is detailed in (3). Coverage shows the provided network coverage by nodes, $\omega_{4}$ and $\omega_{5}$ are predefined constant weights.

#### 4.1.3. Network Changes Optimization Using SA

**Figure 8:**
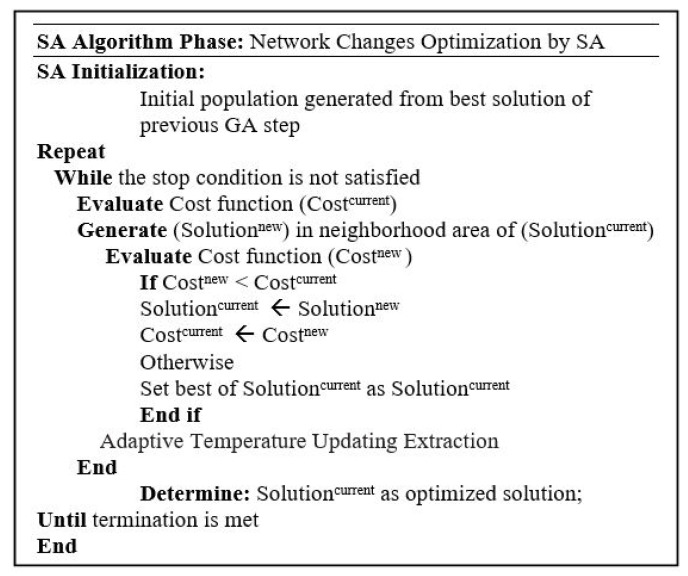
SA Algorithm pseudocode.

Simulated Annealing is a meta-heuristic algorithm that is chosen to perform the network changes, including node addition to, moving in, and removing from the IoT network. Generally, a random primary solution is required to start SA. However, in the proposed hybrid GA-GA-SA approach (Figure 6), the initial solution for SA is selected from the final GA global solution in the previous step. A local search strategy is deployed to improve the network changes by the SA algorithm. A new solution (called $Solution^{new}$) is generated at every iteration of SA that is located in the current solution ($Solution^{current}$) neighborhood area. The case $Cost^{new}<Cost^{current}$ means that the current solution is replaced by the new solution. Otherwise, the new solution can be accepted. The same cost function of the first GA (expression (4)) is considered for SA evaluation at each iteration. Figure 8 shows the SA algorithm.

#### 4.1.4. Clustering Results

In order to study the performance of the proposed clustering algorithms, a network environment for the IoT devices was simulated, as shown in Figure 5. It included 100 nodes randomly generated and distributed in a 2-D network. MATLAB 2018a was employed since it offered a reliable environment for clustering algorithms, facilitated a straightforward simulation of algorithms, so that the results could be ultimately compared. Table 1 provides the GAs parameters deployed in this scenario.

The GAs started from a specific number of individuals, termed population. Each individual in proposed GA algorithms was elevated while using combined cost functions presented in Equations (4) and (5). The network configuration changes were detected by the SA section and then optimized the network accordingly. Table 2 provides the SA parameters used in this scenario.

In the simulation, the energy loss per bit for transmitter or receiver ($E_{elec}$) was considered to be equal to 50 nJ/bit ($E_{elec}$ = 50 nJ/bit), while the constant value for transmission amplifier was $E_{amp}$ = 0.1 nJ/bit/m${}^{2}$ which was in line with the reported work [64].

The proposed clustering algorithm was benchmarked against four following algorithms reported in the literature: ASLPR [60], ERA [65], FSFLA [64], and GAPSO [50].

Application-Specific Low Power Routing (ASLPR) is based on evolutionary algorithms adopted for Wireless Sensor Network (WSN) applications. It uses GA and SA for CH nodes selection. Energy-aware Routing Algorithm (ERA) is for cluster-based WSNs. The residual energy of the CH nodes and the intra-cluster distances is considered in ERA for cluster formation. Fuzzy Shuffled Frog Leaping Algorithm (FSFLA) employs the memetic Shuffled Frog Leaping Algorithm (SFLA) in order to optimize the Mamdani fuzzy rule-base table based on the application specifications. This protocol deals with node energy and intra-cluster distances as well as with network lifetime. Genetic Algorithm and Particle Swarm Optimization (GAPSO) [50] is proposed in order to form clusters in the IoT environment. All of the protocols were evaluated within the same simulated network environment. Each algorithm at the end of its optimization resulted in a different number of clusters and cluster heads.

**Figure 9:**
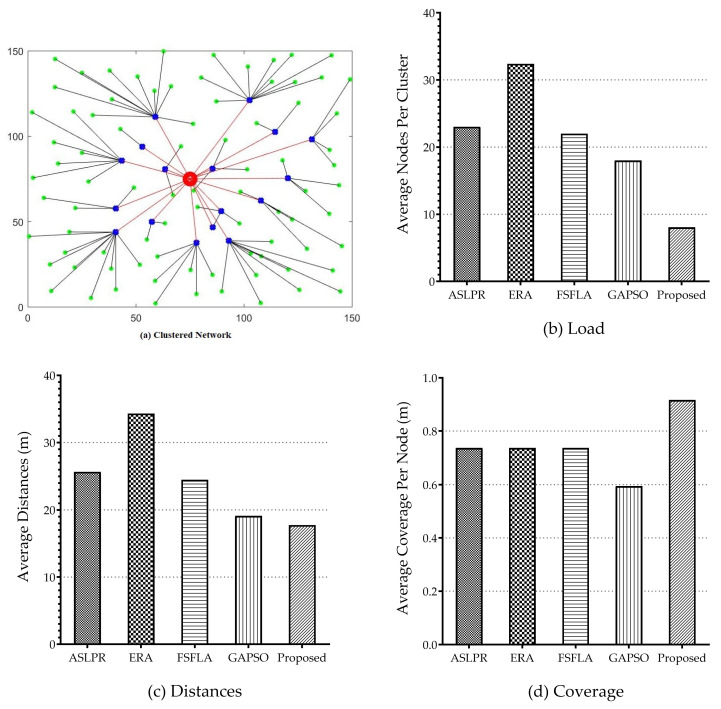
Performance of the proposed clustering algorithm. (**a**): clustered network and CH positions. (**b**–**d**): benchmarked performance in terms of load, distances, and coverage, respectively.

The obtained simulation results indicate the effectiveness of the proposed clustering model as well as the efficiency of the algorithm to minimize the distances and the total network energy. Figure 9a illustrates the formed clusters for GA-GA-SA with the centrally located base station with 100 nodes that were randomly distributed in a network coverage area of 150 (m) × 150 (m). Figure 9b–d show that the proposed GA-GA-SA performs better when compared to the other algorithms by lowering the network load, minimizing the distances, and, therefore, increasing the network coverage.

### 4.2. Blockchain Implementation

#### 4.2.1. Development Environment

We deployed simulation models in two different environments associated with each level of the multi-layer network in order to demonstrate the feasibility and practicability of the proposed blockchain framework. The first model implements the HLF blockchain in Layer-2 encompassing IoT devices, CH nodes (peers), APIs, and an organization. The global blockchain deployment simulator at Layer-3 is conducted to compare both Etherume and HLF metrics implementations. The simulation model at Layer-3 uses a workstation as the BS server running the blockchain applications.

**Figure 10:**
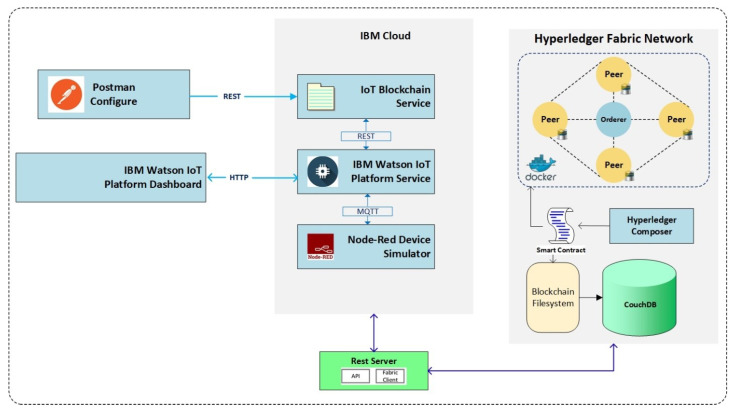
The implementation structure of the blockchain IoT framework.

The Layer-2 implementation environment was created in order tto study the efficiency of the proposed blockchain framework of the multi-layer model, as illustrated in Figure 10. It also shows the means of connection between various entities consisting of IoT devices, IoT server, and blockchain network. The IBM Cloud was used to host development tools and technologies for implementing the IoT devices. IBM Watson IoT Platform [66] was chosen to host IoT devices and gateways. The Node-Red server provided communication between the IoT devices and servers while using the Constrained Application Protocol (CoAP). Physical nodes are simulated in the IBM IoT Watson platform and connected to related Cloud foundry services on the IBM Cloud. The IoT server is organized using a virtual environment that was integrated with various virtual nodes, and a lightweight permissioned HLF blockchain framework is utilized to grant the security for Layer-2. The HLF network within the experimental setup consists of four peers and an orderer node running as docker images using docker containers [67]. The open-source HLF (v1.4) blockchain framework was implemented and hosted by Linux foundations. The Ubuntu Linux 18.4 LTS is the operating system hosted by Intel Core i7-3770 @ 3.4 GHz processor and 16 GB memory. The docker environment is run by the docker engine (version 19.03.8). The configuration of docker images and containers is provided by the docker-compose (version 1.17.0) as the Integrated Development Environment (IDE).

A smart contract was installed and instantiated on peers nodes, and data storage was allocated in order to write a block of transactions to the blockchain ledger. The composer-playground is a web interface for designing and implementing smart contracts and managing transactions and assets. The composer Command Line Interface (CLI) provides an environment to deploy, implement, and execute smart contracts and related definitions by the developers. The peers were set up to use the CouchDB for managing the state data that can handle the complex queries against the transaction logs. The Chain Code (CC) was modelled as JavaScript Object Notation (JSON). The client application can invoke a CC to access the state database and perform various queries, such as put, get, and delete, through APIs. Different blockchain functions were defined by deploying a REST server to directly provide RESTful APIs that can be invoked while using a web client or a virtual device. The user can invoke relevant APIs using GET or POST to submit various transactions through HTTP requests. The REST server hosts the Fabric client application to communicate with the HLF network through google Remote Procedures Calls (gRPC) system deployment. All of the peers in the network have a copy replica of the ledger. The ledger has two parts transaction log and all the recorded state changes. The state data also consists of the key-value pairs that are version. All the state database changes are recorded in time order in the ledger, and the blocks are cryptographically linked together. The orderer node ensures ledger consistency by implementing the PBFT algorithm. The HLF framework supports the Execute-Order-Validate and Commit transaction model.

The Layer -3 simulation model was created while using a workstation as the BS server running the blockchain applications. This environment facilitated measuring the throughput and latency parameters of the Ethereum and Hyperledger private networks. The networks were set up in similar conditions and provided with a virtually generated workload. A distributed environment that includes the two blockchain networks was considered for the experimental set up. The simulation models used a workstation with Intel Core i7-3770 @ 3.4 GHz processor and 16 GB memory as the BS. For simplicity, the Etherume network was deployed with just one mining node. The experimental results are presented in Section 4.3.

#### 4.2.2. Smart Contract for Modeling Transactions

The Hyperledger Composer [68] hosted the blockchain applications and facilitated the design and implementation of smart contracts as well as blockchain applications. A business network was deployed in the Hyperledger Composer through a set of open development tools. The members of business networks were participants. They could submit related transactions. The participants were the owners of IoT devices (CH and related BS nodes) with the management and access abilities for their devices. Assets were services, devices, properties, and goods that were registered and stored within the network. In the reported study, the assets represented IoT devices, including sensors, actuators, or IoT nodes. Each device could be identified through the device ID, device type, device name, device owner, timestamp, event, and value. The presented nodes, including CH ones, were modeled as a different type of assets in the simulation. Transactions represented a logical process within the smart contracts. The implemented model stored the data checksum, data pointers, operations, and ownership of data in the blockchain ledger, while the actual data were held in a separate cloud-server or off-chain storage system. Smart contracts interacted with assets and participants. Besides, a smart contract could set various rules and conditions to perform multiple actions, such as read, create, update, or delete, within the blockchain network. The logical transaction operations were defined in smart contracts as transaction process functions. Smart contracts also included the queries definitions written in a bespoke query language to extract data from the blockchain network. The communication between the blockchain network, IoT device, and the web application was performed by REST APIs that were generated by the composer-rest-server.

### 4.3. Performance Evaluation

The primary objective of a blockchain application is to maintain a number of submitted transactions by the participants. The submitted transactions then proceed to the verification and ordering process, which results in a block generation and storing the transaction outcome on the blockchain ledger. The following metrics are presented by Hyperledger Performance and Scale Working Group [69] to measure the blockchain application performance:

- Transaction Throughput, i.e., the total number of committed transactions by the blockchain System Under Test (SUT) in a given time period in seconds.
- Transaction Latency, i.e., the amount of time that is taken for a transaction to be stored on the blockchain ledger.

The system was tested to evaluate the performance of the proposed model in terms of both the latency and throughput. The results were benchmarked against the parameters reported in the literature with the aim of demonstrating the efficiency of the designed framework. The evaluation was conducted while using the Hyperledger Caliper [70] to facilitate the specific blockchain configuration by the administrator.

In the proposed model, the latency represents the time that is required by CHs to verify new blocks. The block size is an essential factor that affects both node and network latency. The latency is measured by the time that the system requires to reach consensus after the node starts to detect the new block validations. The analysis of the system was conducted with a set of transactions, such as Open, Transfer, and Query. The results were provided for Hyperledger Fabric (proposed blockchain for Layer 2) and Ethereum (standard global blockchain). Table 3 presents the simulation results for evaluating latency and throughput for three different transaction types within HLF implementation. The average latency decreases by implementing a multi-layer model. In this model, only a portion of the nodes (i.e., CHs) is contributing to new blocks validation. Table 3 also presents the Ethereum implementation results. It can be seen that the proposed lightweight HLF blockchain is superior when compared to the Ethereum as a global blockchain technology.

**Figure 11:**
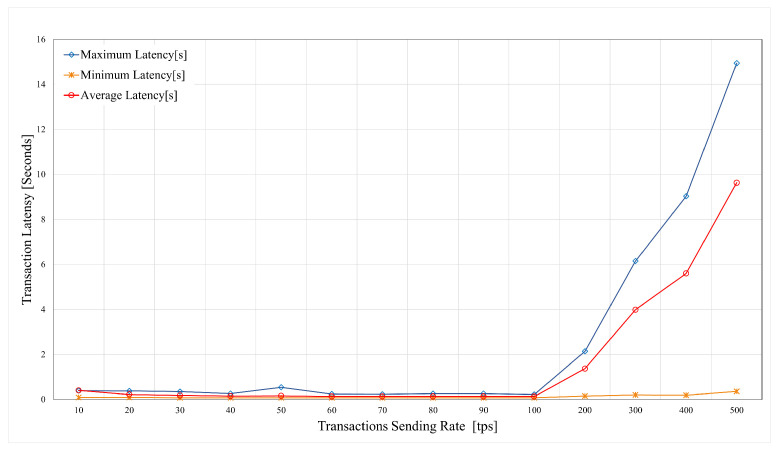
Latency vs. transaction sending rate.

**Figure 12:**
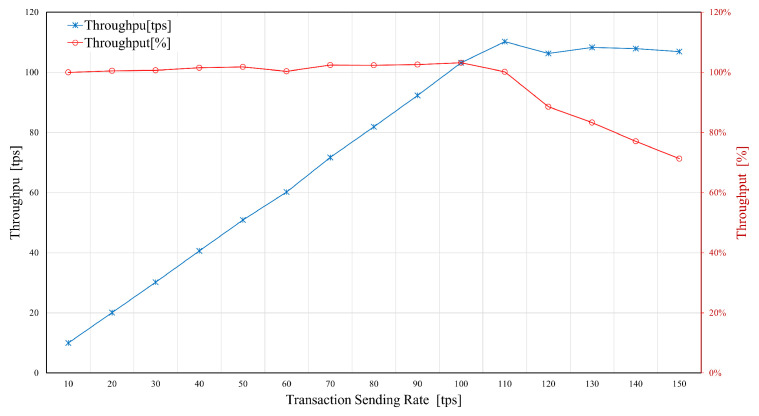
Throughput vs. transaction sending rate.

Despite security and privacy, latency and throughput are essential performance metrics when selecting an appropriate blockchain platform for IoT applications. The resource allocation for the blockchain network must be done in order to meet the latency requirements (for a given input load). A further experiment was conducted to analyze the SUT behavior consisted of multiple rounds of benchmarks with different transaction sending rates. The sending rates varied from 20 to 500 Transactions per Second (TPS), and 1000 transactions were generated for each benchmark to measure the maximum, average, and minimum transaction latency and throughput. Figure 11 presents the maximum, average, and minimum transaction latency for each round of experiments. The minimum latency remained below 1 s during the experiments, while the maximum latency proliferated as the send rate reached the 100 TPS. Figure 12 illustrates the transaction throughput results for varying transaction sending rate. The throughput remained around 100% while the sending rate was up to 110 transactions per second. A significant drop in the throughput was observed as the sending rate increased to 110 TPS, which was the maximum usable sending rate for the SUT.

This experiment only considered an individual client in the blockchain network to generate all the transactions. As expected, the performance of the blockchain network highly depends on the underlying hardware. The HLF provides a three-stage revolutionary architecture known as execute-order-validate, in which every stage depends on previously executed transactions.

Our experiments revealed that the proposed HLF-based blockchain model for IoT application could process up to 110 transactions per second while maintaining a 100% transaction throughput and an average latency of 500 milliseconds, with a maximum of 110 TPS, with throughout that is very close to 100%. A send rate of 100 TPS is sustainable, as the actual throughput is around 100%. However, increasing the send rate to 100 and 200 TPS only yields to a marginal throughput decrease. This lead to the conclusion that our setup can sustain a send rate of about 110 TPS. Therefore, our proposed architecture could support real-time provisioning of multiple 5G-enabled IoT applications without imposing any considerable latency to the process.

The maximum latency grows to nearly 15 s as the number of input transactions increases. This is due to resource restrictions of the containers that are allocated to the peer nodes. The minimum latency remains almost constant, as there are no high loads imposed on the peer nodes at the beginning. Additionally, the blockchain configuration (e.g., the block size, the number of channels, ordering service, users, endorsing nodes) influences the latency. It can be observed that, in all cases, all of the transactions are successfully completed, i.e., no loss of transactions occurs.

## 5. Security Analysis of the Framework

The proposed secured IoT multi-layer model that is based on Hyperledger Blockchain technology offers an overall superiority over the previous works reported in the literature, as illustrated by the metrics comparison that is given in Table 4.

### 5.1. Framework Privacy

Contracts between different entities are recorded in the blockchain system. Therefore, privacy disclosure assessment is required. The identity of an object is encrypted, and the IoT address is recorded in the blockchain as the pseudonym of the entity. The domain name and fixed address for communication are not required, and the blockchain maintains tasks through running smart contracts, as discussed above. In the IoT network, IP address of an object is encrypted and recorded in the blockchain thus leading to the anonymity of the object. The contract context privacy is guaranteed by the Hash Code of the real context within the blockchain network while minimizing the risk of a privacy leak.

### 5.2. Heterogeneity and Flexibility

The proposed framework accommodates various configurations for system security in different scenarios. These include the IoT objects with limited resources, the security of sensitive information, high-risk, and broadcasting. The security configuration options can vary, due to the strength of cryptography techniques and characteristics of key lifetimes (strong crypto, short and long key lifetimes, and lightweight cryptography), key distribution mechanisms, the selection of different session keys, such as encryption and authentication, cached session keys, including of one, multiple, and unlimited keys, different owners of keys, and the stability of the fundamental protocols (TCP and UDP). Besides, a certain degree of flexibility is achievable by granting an option to a node or entity to connect or leave the system freely. Changes in the network are recorded in the blockchain through the distributed consensus process.

### 5.3. Authentication

The process of authentication is implemented in two parts: (1) local authentication and authorization process in the infrastructure layer and (2) rights to objects by smart contracts. The node requirements and respective rights are recorded in the blockchain that was implemented in different segments. The block summary consists of a contract summary. It is accessible at any time. The non-repudiation nature of this summary guarantees the interests of the object.

The multi-layer approach through the network clustering divides the entire IoT network into different tiers, as presented in Figure 2. This includes the local authentication services and globally distributed blockchain-based framework, while separating the external authority. Therefore, the effect of a local authentication service failure or attack to the network is limited to the compromised nodes, while the impact on the network is significantly reduced.

### 5.4. Scalability

The framework tackles the following scalability challenges: (1) high data traffic and (2) a massive number of IoT objects and devices. The multi-layer structure facilitates multiple cluster implementation and fulfills the scalability issues. Two different CH nodes can establish different secure communications on a client-server basis. CH establishes secure communication with the entities within the same cluster in order to avoid the overhead incrementation. When networked CH nodes start communicating within a framework that is based on the blockchain, the exchange of cryptographic keys is necessary before beginning the client-server communication by which further overhead is reduced.

## 6. Conclusions

This paper proposes a multi-layer security model for IoT devices functioning under multi-hop cellular networks based on distributed technology of the blockchain. The developed model provides a feasible solution to establish the decentralized application of the blockchain technology for the security of the cellular-enabled IoT network. The hybrid self-clustering EC algorithm, utilizing GA and SA, is developed to fragment the IoT network into clusters in order to provide the multi-layer structure and enhance the network lifetime. Detailed system implementation is discussed, and the way the blockchain-based model can help to improve the IoT system authentication and authorization is elaborated. The model proposes the open-source HLF blockchain for deployment and verification. The multi-layer model enhances network security, lowers the processing load, and reduces network load and latency. The proposed implementation enhances the efficiency of the communications via the peer-to-peer nature of the blockchain communication and maps it to the device-to-device communication in cellular systems with improved integrity and security. The proposed solution tackles the IoT security challenges, including framework privacy, authentication, heterogenicity, and flexibility, as well as network scalability. The proposed hybrid clustering algorithm has been compared with four existing protocols. The simulations study demonstrates that the proposed algorithm outperforms the competitors in terms of various performance metrics, including network load, network coverage, and distances. The performance of the proposed multi-layer blockchain-based framework was evaluated. It was found that the lightweight blockchain was more effective than the global blockchain Ethereum.

The focus of our future work will be on the deployment of a practical scalable test-bed configured as MBS framework of IoT devices to study, analyze, and compare the performance in the real world environment.

## Figures and Tables

**Table 1:** GAs Parameter Settings.

GAs Parameters	Value
Population Size	30
Selection Type	Proportional Selection
Recombination Percentage	0.1
Crossover Percentage	0.5
Crossover Type	One-Point
Mutation Percentage	0.4
Mutation Rate	0.05
Generation Size	500

**Table 2:** SA Parameter Settings.

SA Parameters	Value
Max Iter SA	1000
T initial	0.001
T final	0.000
Pchange Max	0.05
Pchange Min	0.02

**Table 3:** Hyperledger and Ethereum performance metric summary (H: Hyperledger Fabric, E: Ethereum).

Name	Send Rate (TPS)		Max Latency (s)		Min Latency (s)		Avg Latency (s)		Throughput (TPS)	
Blockchain	H	E	H	E	H	E	H	E	H	E
Open	20.2	22.7	0.38	7.05	0.04	2.12	0.18	4.58	20.1	10
Query	10	10.2	0.07	0.02	0.01	0.01	0.01	0.01	10	10.2
Transfer	10	10.7	0.38	7.13	0.06	2.07	0.19	4.63	10	6.7

**Table 4:** Security challenge comparison of blockchain applications in IoT systems.

Ref	IoT Application	Security Challenges				Implemented Consensus	Implemented Blockchain
		Framework Privacy	Heterogeneity and Flexibility	Authentication	Scalability		
[38]	Smart Grids, Smart Cities		Yes		Yes	PoW	Private
[71]	Microgrids, Smart Grids, Vehicle-to-Grids	Yes				PoW	Consortium
[72]	Microgrids, Smart Grid	Yes				PoC	Private
[73]	Big Data, eHealth	Yes			Yes	PoW	Public
[74]	Industrial IoT	Yes	Yes			PoW	Private
[75]	Smart Factory, Supply Chain		Yes			PoS	Consortium
[76]	Industrial IoT, Energy Harvesting networks	Yes	Yes			PoW	Consortium
[77]	eHealth	Yes				PoW	Public
[78]	Mobile edge computing, eHealth		Yes		Yes	PoC	Permissioned
[79]	Cloud computing, V2X	Yes		Yes		PoS	Consortium
[80]	Vehicular Edge Computing		Yes	Yes		PoW	Consortium
proposed	5G MBS	Yes	Yes	Yes	Yes	PBFT, PoC	Consortium

## Data Availability

The data presented in this study are available on request from the corresponding author.

## Abbreviations

The following abbreviations are used in this manuscript:

ASLPR	Application-Specific Low Power Routing
BCS	Blockchain Structures
BS	Base Station
CC	ChainCode
CH	Cluster Head
CLI	Command Line Interface
CoAP	Constrained Application Protocol
D2D	Device-to-Device
EC	Evolutionary Computation
ECC	Elliptic Curve Cryptography
ERA	Energy-aware Routing Algorithm
FSFLA	Fuzzy Shuffled Frog Leaping Algorithm
GA	Genetic Algorithm
GAPSO	Genetic Algorithm and Particle Swarm Optimization
gPRC	google Remote Procedures Calls
HLF	Hyperledger Fabric
IDE	Integrated Development Environment
IoT	Internet of Things
LSB	Lightweight Scalable Blockchain
LHB	Lightweight Hyperledger Blockchain
LTS	Long Term Support
MCNs	Multihop Cellular Networks
MSP	Membership Service Providers
OS	Ordering Service
PKI	Public Key Infrastructure
PoBT	Proof of Block and Trade
PoC	Proof of Concept
PoW	Proof of Work
RoA	Rating of Allocation
SA	Simulated Annealing
SDK	Software Development Kit
SDN	Software Defined Networking
SFLA	Shuffled Frog Leaping Algorithm
SI	Swarm Intelligence
SUT	System Under the Test
TLS	Transport Layer Security
TSP	Transactions Per Second
